# Advanced nasopharyngeal carcinoma: pre-treatment prediction of progression based on multi-parametric MRI radiomics

**DOI:** 10.18632/oncotarget.19799

**Published:** 2017-08-02

**Authors:** Bin Zhang, Fusheng Ouyang, Dongsheng Gu, Yuhao Dong, Lu Zhang, Xiaokai Mo, Wenhui Huang, Shuixing Zhang

**Affiliations:** ^1^ Medical Imaging Center, First Affiliated Hospital of Jinan University, Guangzhou, P.R. China; ^2^ Institute of Molecular and Functional Imaging, Jinan University, Guangzhou, P.R. China; ^3^ Department of Radiology, The First People's Hospital of Shunde, Foshan, P.R. China; ^4^ Key Laboratory of Molecular Imaging, Chinese Academy of Sciences, Beijing, P.R. China; ^5^ Department of Radiology, Guangdong General Hospital/Guangdong Academy of Medical Sciences, Guangzhou, P.R. China

**Keywords:** imaging, biomarkers, nasopharyngeal carcinoma, progression, radiomics

## Abstract

We aimed to investigate the potential of radiomic features of magnetic resonance imaging (MRI) to predict progression in patients with advanced nasopharyngeal carcinoma (NPC). One hundred and thirteen consecutive patients (01/2007-07/2013) (training cohort: n = 80; validation cohort: n = 33) with advanced NPC were enrolled. A total of 970 initial features were extracted from T2-weighted (T2-w) (n = 485) and contrast-enhanced T1-weighted (CET1-w) MRI (n = 485) for each patient. We used least absolute shrinkage and selection operator (Lasso) method to select features that were most significantly associated with the progression. The selected features were used to construct radiomics-based models and the predictive performance of which were assessed with respect to the area under the curve (AUC). As a result, eight features significantly associated with the progression of advanced NPC were identified. In the training cohort, a radiomic model based on combined CET1-w and T2-w images (AUC: 0.886, 95%CI: 0.815-0.956) demonstrated better prognostic performance than models based on CET1-w (AUC: 0.793, 95%CI: 0.698-0.889) or T2-w images alone (AUC: 0.813, 95%CI: 0.721-0.904). These results were confirmed in the validation cohort. Accordingly, MRI-based radiomic biomarkers present high accuracy in the pre-treatment prediction of progression in advanced NPC.

## INTRODUCTION

Nasopharyngeal carcinoma (NPC) is a rather common malignant tumor among Asians, especially the South China [1]. Radiotherapy (RT) is regarded as the standard treatment for patients with NPC. Up to now platin-based radiochemotherapy has been established in the treatment of NPC, survival rates have been improved [2]. For patients with advanced NPC (stage III-IVb), their prognosis are poorer due to treatment failure. The main causes of treatment failure are locoregional recurrences and distant metastasis [3]. Pretreatment prediction of recurrence and distant metastasis is crucial to make decisions regarding treatment. If poor survival can be predicted prior to treatment, then this will help to determine whether more aggressive treatments should be administered, such as, by increasing cycles, or by using of adjuvant and/or induction chemotherapy.

Tumor-node-metastasis (TNM) indicates tumor extent (T), lymph node metastsis and its extent (N), and distant metastasis (M) [4]. Although the TNM staging system for NPC plays a crucial role in predicting prognosis and facilitate treatment stratification, it may not be sufficiently precise; indeed, patients with the same TNM stage often have different survival times. Thus, new tools are urgently needed to identify patients who are at risk of having a poor prognosis.

The emergence of radiomics has broaden the scope of routine medical imaging in clinical oncology [5]. By converting medical images into high-dimensional, mineable, and quantitative features via high-throughput extraction of data-characterization algorithms, radiomics provides an unprecedented opportunity to improve decision- support in oncology at low cost and noninvasively [6, 7]. It hypothesizes that medical imaging can reveal crucial information regarding tumor phenotype [8]. The imaging features are extracted from entire tumors, and hence are likely to characterize the intra-tumor heterogeneity. It has been reported that intra-tumor heterogeneity could have profound significance in clinical practice, such as cancer diagnosis, staging, prognosis, prediction and response to treatment [9]. Therefore, it is regarded as a very important factor for precision oncology. Several studies have investigated the potential of radiomics in a range of cancer types and modalities (e.g. CT, MRI, and PET/CT). It have been proved that radiomic features are associated with tumor grades, stages, patient survival, and other clinical outcomes [10–13]. Fox example, patients with stage III-IV primary colorectal cancer had significantly higher CT-based Rad-score than those patients with stage I-II. Prognostic characteristics of radiomic features are cancer-specific and can be used for building prognostic models. Computer-extracted magnetic resonance (MR) image-based tumor phenotypes can be predictive of the molecular classification of invasive breast cancers. These previous studies indicated that radiomics could affect individualized treatment strategy and monitor the clinical process. Therefore, radiomics is a novel and promising step toward the realization of personalized cancer care.

To our knowledge, no recent studies have investigated whether the prognosis of NPC could be predicted by radiomics-based prognostic models. Thus, in this study, we developed and validated multiparametric MRI-based radiomic signature as a novel biomarker for providing individualized, pretreatment predictions of progression in patients with advanced NPC (TNM stage: III-IVb).

## RESULTS

### Clinical characteristics of the patients

The clinical characteristics of the training and validation cohorts are shown in Table 1. No differences were found between the training and validation cohorts in terms of age, gender, overall stage, T-stage, N-stage, histology, or follow-up time (*p* = 0.076-0.941). The median follow up time was 39 months (range, 3-89 months).

**Table 1:** Patient and tumor characteristics in the training and validation cohorts

	Training cohort(N = 80)	Validation cohort(N = 33)	p-value
Gender			
Male	62 (77.5%)	25 (75.8%)	0.841
Female	18 (22.5%)	8 (24.2%)	
Age (years)			
Median (IQR)	42.5 (37-51.00)	43.5 (35.3-51.3)	0.370
< 40	33 (41.3%)	13 (39.4%)	
40-50	24 (30%)	11 (33.3%)	0.941
>50	23 (28.8%)	9 (27.3%)	
Overall stage			
III	50 (62.5%)	23 (69.70%)	0.467
IV	30 (37.5%)	10 (30.3%)	
T stage			
T1	3 (3.75%)	4 (12.1%)	0.153
T2	20 (25.0%)	4 (12.1%)	
T3	38 (47.5%)	19 (57.6%)	
T4	19 (23.8%)	6 (18.2%)	
N stage			
N0	7 (8.8%)	1 (3.0%)	0.384
N1	17 (21.3%)	6 (18.2%)	
N2	43 (53.8%)	23 (69.7%)	
N3	13 (16.3%)	3 (9.1%)	
Histology*			
WHO type I	0	0	---
WHO type II	1 (1.3%)	5 (15.2%)	
WHO type III	79 (98.8%)	28 (84.9%)	
Follow-up time (mo)			
Median (IQR)	39 (25.3-69)	39.5 (28.5-50.3)	0.076

Data are n (%) unless otherwise indicated. *Histology was categorized according to the WHO Classification. IQR: inter-quartile range; type I: keratinizing; type II: non-keratinizing differentiated; type III: non-keratinizing undifferentiated.

### Radiomic feature extraction/selection and radiomic model building

A total of 970 features were extracted from magnetic resonance images (485 features from T2-w images and the remaining 485 from CET1-w images). Of these, we selected five textural features (i.e. CET1-w_5_fos_median, CET1-w _5_GLRLM_RP, CET1-w_5_GLCM_correlation, CET1-w_5_GLRLM_ SRE CET1-w_4_ GLRLM_ LRHGLE) from CET1-w images and six features (i.e. T2-w_Max3D, T2-w_4_fos_ mean, T2-w_7_fos_mean, T2-w_5_GLCM_sum_average, T2-w_6_GLCM_IMC1, T2-w_1_GLRLM_SRLGLE) from T2-w images that were most strongly associated with the outcome progression of advanced NPC in the training cohort. To build the radiomics-based prognostic models, eight features were selected for inclusion in the Rad-score predictive model, including four features derived from CET1-w images and four features derived from T2-w images. Rad-score calculation formula was as follows:

Rad-score = 0.0330481732 CET1-w_5_fos_median
-6.4931353700 CET1-w_1_GLCM_correlation
-0.0008289514 CET1-w_4_GLRLM_LRHGLE
+ 9.7275394149 CET1-w_5_GLRLM_RP
+ 0.0106439280 T2-w_Max3D
-0.1787872430 T2-w_4_fos_mean
-0.4498668025 T2-w_7_fos_mean
+ 0.1613474592 T2_5_GLCM_sum_average
+ 65.1061061821

### Prognostic performance of radiomic models

**Figure 1:**
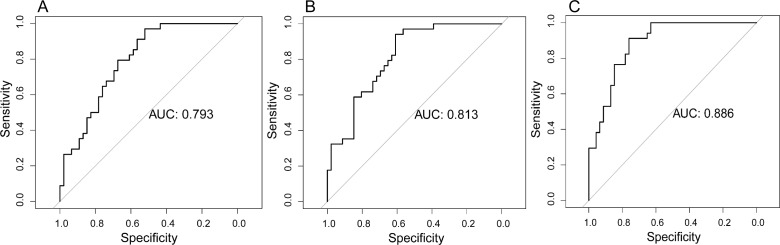
Prognostic performance of radiomic models in the training cohort **(A)** Radiomic model based on CET1-w images. **(B)** Radiomic model based on T2-w images. **(C)** Radiomic model based on joint CET1-w and T2-w images.

In the training cohort, the radiomics model based on CET1-w images yielded an AUC of 0.793 (95%CI: 0.698 to 0.889). The radiomic model based on T2-w images yielded an AUC of 0.813 (95% CI: 0.721 to 0.904). The radiomic model from joint CET1-w and T2-w images yielded the highest AUC, which was 0.886 (95% CI: 0.815 to 0.956) (Figure 1).

**Figure 2:**
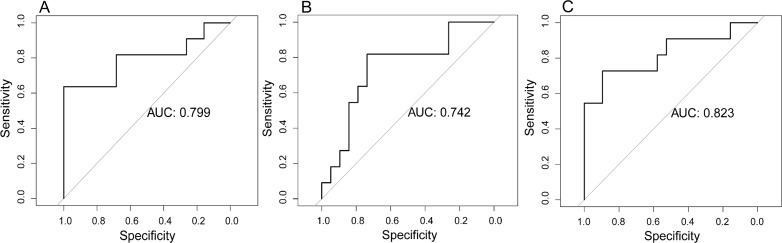
Prognostic performance of radiomic models in the validation cohort **(A)** Radiomic model based on CET1-w images. **(B)** Radiomic model based on T2-w images. **(C)** Radiomic model based on joint CET1-w and T2-w images.

In the validation cohort, the radiomic model based on CET1-w images achieved an AUC of 0.799 (95% CI: 0.602 to 0.996). The radiomic model based on T2-w images achieved an AUC of 0.742 (95% CI: 0.548 to 0.935). The radiomic model based on joint CET1-w and T2-w images achieved the highest AUC, which was 0.823 (95% CI: 0.645 to 1.000) (Figure 2).

### Group differences

**Figure 3:**
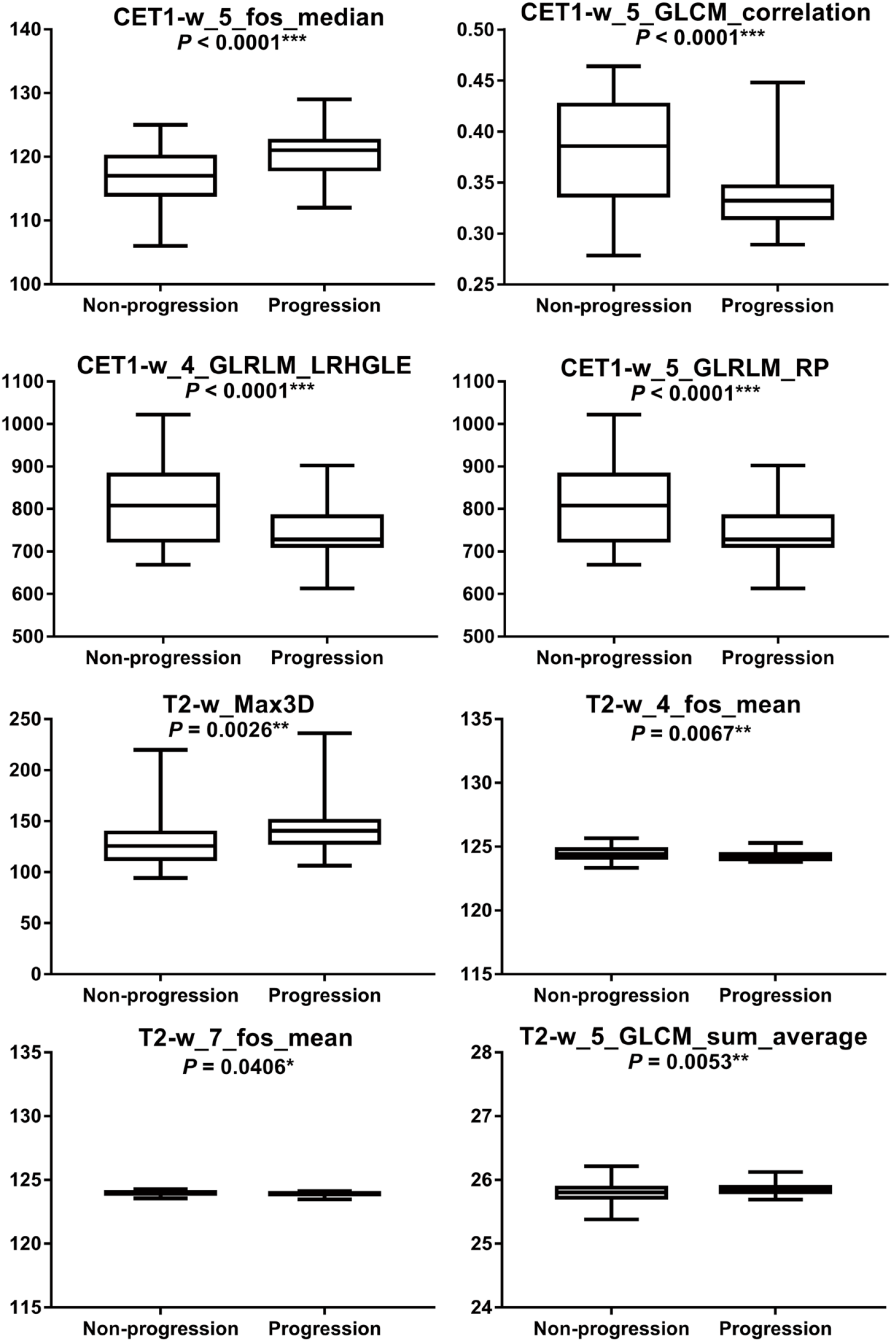
Boxplotsregarding statistical differences between progression group and non-progression group were shown * Indicates statistically significant.

The eightfeatures selected by Lasso model (i.e. CET1-w_5_fos_median, CET1-w_5_ GLCM_ correlation, CET1-w_4_GLRLM_LRHGLE, CET1-w_5_GLRLM_RP, T2-w _4_fos_mean, T2-w_Max3D, T2-w_7_fos_mean, and T2-w_5_GLCM_sum_average) fromCET1-w and T2-w images showed significantly differentbetween progression group and non-progression group with p-values < 0.05, especially for the first four features (p < 0.001, for all) (Figure 3).

## DISCUSSION

In the present study, a novel 8-feature based Rad-score was developed and validated to be an independent predictor of progression of advanced NPC. We found the radiomic model from joint CET1-w and T2-w images had better prognostic performance than that from either CET1-w or T2-w images alone. The AUC for the combined model was as high as 0.886 in the training cohort and 0.823 in the validation cohort. Therefore, we identified multi-parametric MRI-based radiomics as a new biomarker for prognostic prediction in advanced NPC.

Although the tumor-node-metastasis (TNM) staging system for NPC plays a crucial role in predicting prognosis and facilitate treatment stratification, it may not be sufficiently precise [14]. The traditional TNM staging system is based merely on gross anatomy, it has an obvious limitation that intra-tumor heterogeneity is not concerned. Intra-tumor heterogeneity has been reported to have profound significance in clinical practice, such as diagnosis, staging, prognosis, and thus it is regarded as a very important factor for precision oncology. This view fits our current knowledge of cancer, in which malignant lesions consist of heterogeneous cell populations with distinct molecular and micro-environmental differences. Hence the current interest in using medical imaging to repetitively assess intra-tumor spatial and temporal heterogeneity. Radiomics is a new and promising area of research in the field of imaging with tremendous potential to unravel the hidden information in medical images [15]. The radiomic features are extracted from entire tumors on medical images, and hence are likely to characterize the intra-tumor heterogeneity. Radiomics is based on imaging, but beyonds imaging. It has been demonstrated to benefit the field of oncology by assessing the influence that pre-treatment tumor properities, and post-treatment effects on the texture and intensities of the affected tissues [16–18]. Yong et al. identified prognostic intratumor heterogeneity using pre- and post-RT 18F-FDG PET textures for pancreatic cancer patients. They found that most of the post-RT features were significant with the PET response, whereas clincal stage was not associated with the response [19]. Huang et al. found incorporating the radiomics signature into the radiomics-based nomogram resulted in better performance (p < 0.0001) for the estimation of disease-free survival (DFS) (C-index: 0.72) than with the clinical-pathologic nomogram (C-index: 0.691) [20]. Yuan et al. reported the radiomic tumour-phenotypes biomarker exhibited better diagnostic accuracy than traditional volumetric analysis in discriminating lung adenocarcinoma with different disease- specific survival [21]. It is accepted that radiomics can act as a guide in the disease or cancer diagnosis, grading, staging, monitoring patients on therapy, predicting treatment response, and determine patient outcomes [13, 22–28]. Since the emergence of radiomics, a large amounts of quantitative features can now be extracted from routine medical images through high-throughput computing algorithms, and these can be converted into mineable data that contributing to associating imaging phenotypes with clinical data, genomics, proteomics, and other “omics” information [15, 29].

MRI is routinely used to diagnose and monitor NPC. Unlike CT, MRI provides better tissue contrast, has multiplanar capacity, and exhibits fewer artifacts from radiation and bone beam hardening. Therefore, we extracted radiomic features from multiparametric MR images including T2-w and contrast-enhanced T1-w images. We found that the radiomic model derived from combined T2-w and contrast-enhanced T1-w images outperformed model derived from T2-w or contrast-enhanced T1-w images alone in both the training cohort and validation cohort.

To develop the radiomics-based prognostic models, 970 candidate features were reduced to a set of only eight potential prognostic factors by using a Lasso logistic regression model. Lasso is suitable for analyzing large sets of radiomic features with a relatively small sample size, and it is designed to avoid overfitting [30]. The radiomic features obtained from Lasso are generally accurate, and the regression coefficients of most features are shrunk towards zero during model fitting, making the model easier to interpret and allowing the identification of imaging features that are most strongly associated with tumor progression [31]. The eight most powerful radiomic features showed significantly different between progression group and non-progression group. We then used the eight radiomic features weighted by their coefficients to build the radiomic models. Using a 10-fold cross-validated design, the top-performing logistic regression model yielded an AUC = 0.886.

The limitations to this study included the fact that our analysis did not account for two-way or higher-order interactions between features. If interactions between features had been identified, the interaction terms that were most strongly associated with the outcome interactions would have been selected when we constructed the radiomics score, and this could have improved predictive performance. We used a validation cohort that was drawn from the same institution as the training cohort, which prevented us from investigating the generalizability of the results to other institutions and settings.

In summary, the present study developed and validated multiparametric MRI- based radiomics as novel biomarkers to predict progression pre-treatment in patients with advanced NPC (TNM stage: III-IVb). Radiomics-based prognostic models could potentially be useful for precision oncology and affect the treatment strategies that are used for patients with NPC. Larger studies are needed to prospectively explore the prognostic performance of textural and non-textural MRI-based radiomic features as noninvasive predictors of NPC progression. Associations between radiomic features and clinical data, genomics, proteomics are also warranted to investigate in the future.

## PATIENTS AND METHODS

### Patients

Our Institutional Review Board approved this retrospective study and waived the need to obtain informed consent from the patients. We reviewed the medical records from the January 2007 to July 2013 to identify patients who had histologically confirmed NPC (TNM stage: III-IVb). Tumor staging was performed according to the American Joint Committee on Cancer TNM Staging System Manual, 7th Edition [13]. All patients underwent pre-treatment 3.0 T MRI scans (Discovery MR 750 System; GE Healthcare, Milwaukee, WIS). The inclusion criteria were as follows: inclusion criteria included (a) Patients with histologically confirmed nNPC (without evidence of recurrence or distant metastases at diagnosis. (b) First MRI images (including CET1-w and T2-w images) before treatment were available. (c) All patients were followed up every 1-3 months during the first 2 years, every 6 months in years 2-5, and annually thereafter. (d) All local recurrences were diagnosed by flexible nasopharyngoscopy and biopsy and/or MRI scans of the nasopharynx and skull base that showed progressive bone erosion and/or soft tissue swelling. Regional recurrences were diagnosed by clinical examination of the neck and, in doubtful cases, by fine-needle aspiration or MRI scans of the neck. Distant metastases were diagnosed based on clinical symptoms, physical examination, and imaging methods including chest X-ray, whole-body bone scan, MRI/CT, PET/CT, and abdominal sonography. (e) Clinical data were available, such as for age, gender, histology, overall stage.

A total of 113 consecutive patients met the criteria (87 men and 26 women; mean age, 43 years ± 11.1) were identified and divided into two cohorts (training cohort: n = 80; validation cohort: n = 33) using computer-generated random numbers. Eighty patients were allocated to the training cohort (62 men and 18 women; mean age, 43 years ± 10.9), while 33 patients were allocated to the validation cohort (25 men and 8 women; mean age, 43 years ± 11.8).

### Clinical endpoint

We chose the progression as the clinical endpoint. Locoregional recurrences or distant metastases were regarded as disease progression. We dichotomized the censored continuous progression-free survival data using a cutoff time of 3 years. Patients who progressed within the cutoff time were labeled as 1, whereas the patients did not progress within the cutoff time were labeled as 0.

### Overview

**Figure 4:**
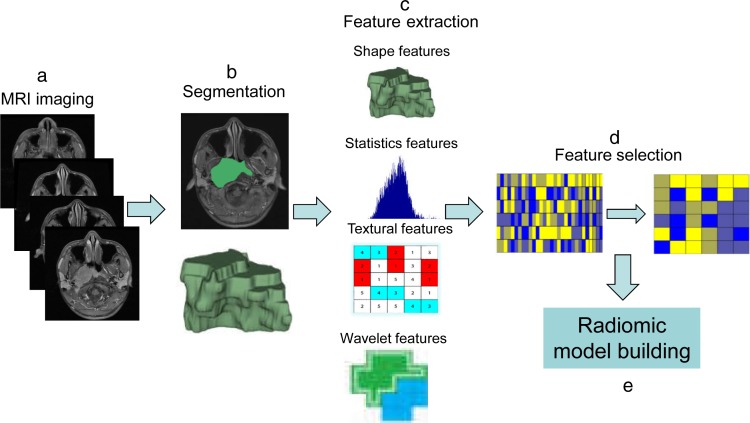
The workflow of radiomics **(a)** MRI imaging. **(b)** Image segmentation was performed on contrast-enhanced T1-w images and T2-w MRI images. Experienced radiologists contour the tumor areas on all MRI slices. **(c)** Features are extracted from within the defined tumor regions, quantifying tumor intensity, shape, texture, and wavelet filter. **(d)** Feature selection by Lasso. **(e)** Radiomic model building.

The proposed noninvasive NPC progression estimation method consists of MRI imaging, image segmentation, high-throughput feature extraction, feature selection and radiomic model building. The radiomics workflow was illustrated in Figure 4. Details of each procedure are described below.

### MRI acquisition and segmentation

We used axial T2-weighted (T2-w) Digital Imaging and Communications in Medicine (DICOM) images and contrast-enhanced T1-weighted (CET1-w) DICOM images that had been archived in the Institutional Picture Archiving and Communication System (PACS, Carestream, Canada), without applying any preprocessing or normalization. The MRI acquisition parameters were as follows: axial T2-w images (TR/TE: 5000/ 85 msec, FOV = 23 × 23 cm, NEX = 2.0, Slice thickness = 4 mm, Spacing = 1.0 mm) and axial CET1-w images (TR/TE: 410/min full msec, FOV = 23 × 23 cm, NEX = 2.0, Slice thickness = 4 mm, Spacing = 1.0 mm).

We used ITK-SNAP software for three-dimensional manual segmentation (open source software; http://www.itk-snap.org). All manual segmentations of the tumor were performed by a radiologist who had 10 years of experience, and each segmentation was validated by a senior radiologist, who had 20 years of experience in NPC diagnosis. The region of interest covered the whole tumor and was delineated on both the axial T2-w images and CET1-w images on each slice.

### Radiomic feature extraction/selection and radiomic signature building

Radiomic features were divided into four types, including first-order statistics features (n = 17), shape- and size-based features (n = 8), statistics-based textural features (n = 36), and wavelet features (n = 424). The feature extraction methodology has been described in the Supplementary Materials. All feature extraction methods were implemented using MatLab 2014a (MathWorks, Natick, MA, USA). The least absolute shrinkage and selection operator (Lasso) logistic regression was used to select the most powerful predictive features associated with the progression from the training cohort. Radiomics signature were built using radiomics score (Rad-score). The Rad-score was calculated for each patient as a linear combination of selected features that were weighted by their respective coefficients.

### Validation of the performance of radiomic models

The predictive performance of the radiomic models were assessed in the training cohort and then tested in the validation cohort using the AUC, along with the 95% confidence interval (CI).

### Statistical analysis

The statistical analyses were performed with R software (R Core Team. R: A language and environment for statistical computing. R Foundation for Statistical Computing, Vienna, Austria. URL: http://www.R-project.org). The package ‘glmnet’ was used for Lasso logistic regression. Differences between progression group and non-progression group with respect to selected features were compared using t test. All statistical tests were two-sided, and p-values of < 0.05 were considered significant.

## SUPPLEMENTARY MATERIALS



## Abbreviations

NPC: nasopharyngeal carcinoma
MRI: magnetic resonance imaging
Lasso: least absolute shrinkage and selection operator
AUC: area under the curve
CI: confidence interval
FOV: field of view
NEX: number of excitations
TR: repetition time
TE: echo time
CET1WI: contrast-enhanced T1-weighted imaging
T2WI: T2-weighted imaging
GLCM: Gray-level co-occurrence matrix
GLRLM: Gray-level run-length texture matrix
