# Supplementary material for: Advanced nasopharyngeal carcinoma: pre-treatment prediction of progression based on multi-parametric MRI radiomics
Source: Oncotarget. 2017 Aug 2;8(42):72457–65. doi: 10.18632/oncotarget.19799 (PMC5641145; doi:10.18632/oncotarget.19799)
Supplement: Supplementary file 1 [file oncotarget-08-72457-s001.pdf]

Advanced nasopharyngeal carcinoma: pre-treatment prediction of progression based on multi-parametric MRI radiomics

SUPPLEMENTARY MATERIALS

Radiomics feature extraction methodology

In this study, a total of 970 candidate radiomics features were generated, of which 485 features were from CETI-w images and the remaining 485 were from T2-w images. All feature extraction methods were implemented using MatLab 2014a (MathWorks, Natick, MA, USA). The 485 features were divided into four types: first-order statistics features, shape- and size-based features, statistics-based textural features, and features after wavelet transform.

First-order statistics features

First-order statistics describe the distribution of voxel intensities within the MRI image through commonly used and basic metrics. To analyze the spatial distribution of the pixels' hue matrix and extract static features of images, a fuzzy similitude matrix is defined. The matrix describes the image's feature space. Seventeen first-order statistics features were used, such as energy, entropy, skewness, kurtosis, mean, maximum, and minimum.

Shape- and size-based features

In this group of features, we included eight descriptors of the three-dimensional size and shape of the tumor region. They included surface area, volume, surface-to-volume ratio, maximum three-dimensional diameter, sphericity, spherical disproportion, and compactness 1 and 2.

Statistics-based textural features

Textural features are visual characteristics that reflect the homogeneity phenomenon of images and the arrangement of properties that change slowly or periodically on the body surface. Our textural features mainly included two typical matrices: the Gray-level co-occurrence matrix (GLCM) and the Gray-level run-length texture matrix (GLRLM). GLCM is the matrix function that describes the distance and angle of each pixel. By calculating the correlation between two gray levels with certain directions and distances, GLCM can reflect integrated information regarding the direction, interval, amplitude, and frequency of images. Gray-level run length metrics (GLRLM) quantify gray level runs in an image. A gray level run is defined as the length (number of consecutive pixels) that have the same gray-level value. We extracted 22 radiomics features from the GLCM and 14 features from the GLRLM. The radiomics features in the GLCM mainly consisted of energy, entropy, correlation, contrast, homogeneity, autocorrelation, mean, variance, dissimilarity, and angular second moment. The radiomics features in the GLRLM mainly consisted of features such as run length non-uniformity, short/long run emphasis, and Gray level non-uniformity.

Wavelet features

The undecimated three-dimensional (3D) wavelet transform was used to decompose the original image, which can be regarded as a preprocessing prior to feature extraction. By changing the ratio of high-frequency to low-frequency signal in images, wavelet transform increases the information of low-frequency signal. Consider L and H to be a low-pass and high-pass functions respectively, X to be the decomposing image, and the wavelet decompositions of X to be labeled as X_{LLL}, X_{LLH} X_{LHL}, X_{LHH}, X_{HLL}, X_{HLH}, X_{HHL}, X_{HHH}. Then, we can obtain eight new images which are decomposed in three directions (x, y, z). The size of each decomposition is equal to the original image and each decomposition is shift invariant. For each decomposition, we computed the first-order statistics and textural features described above. This resulted in 424 features. In the end, we extracted 485 features for each of the MRI series.

Using a least absolute shrinkage and selection operator (LASSO) algorithm, we selected the eight features that were most associated with PFS. The eight features were as follows: ***CET1-w_1_GLCM_energy, CET1-w_4_GLRLM_LRHGLE, CET1-w_5_fos_median, CET1-w_5_GLCM_correlation, CET1-w_5_GLRLM_RP, T2-w_Max3D, T2-w_3_fos_mean,*** and ***T2-w_4_fos_mean.***

As we can see, all features were from images decomposed by undecimated three-dimensional wavelet transforms. The first five features in T1-w images were composed of X_{LLL} (marked by "1"), X_{LHH} (marked by "4"), and X_{HLL} (marked by "5") images. Similarly, the ***T2-w_3_fos_mean*** was X_{LLH} (marked by "3") and the ***T2-w_4_fos_mean*** was the X_{LHH} image feature extracted from T2-w images. In addition, the T2-w_Max3D was the original image feature extracted from T2-w images. In our subsequent discussion, we let X denote the three-dimensional image matrix with N voxels in the analysis of

the first-order statistics features and shape- and size-based features that had been selected by the LASSO algorithm. At the same time, we considered the GLCM and GLRLM to be a matrix with size, which was defined as $P(i,j;\delta,\alpha)$. Here, the (i, j) element represents the number of times the combination of intensity levels appears in two pixels in the image that are separated by a distance of δ pixels in direction α, is the number of discrete gray level intensities, and is the number of different run lengths. Detailed explanations of these terms are provided below:

CET1-w_1_GLCM_energy

The energy of the whole element in the GLCM matrix for an arbitrary δ and α:

$$energy=\sum_{i=1}^{N_g}\sum_{j=1}^{N_g}[P(i,j)]^2$$

CET1-w_4_GLRLM_LRHGLE

The long run high gray level emphasis in the gray-level run-length matrix of textural features in the X_{LHH} images:

$$LRHGLE=\frac{\sum_{i=1}^{N_g}\sum_{j=1}^{N_r}\left[\frac{p(i,j|\alpha)j^2}{i^2}\right]}{\sum_{i=1}^{N_g}\sum_{j=1}^{N_r}p(i,j|\alpha)}$$

CET1-w_5_fos_median

The first-order statistics feature that describes the median value of the intensity levels in CET1-w images in the X_{HLL} images.

CET1-w_5_GLCM_correlation

The correlation in the GLCM that describes the degree of similarity of the matrix elements in a row or column direction in the X_{HLL} images:

$$correlation=\frac{\sum_{i=1}^{N_g}\sum_{j=1}^{N_g}ijP(i,j)-\mu_i(i)\mu_j(j)}{\sigma_x(i)\sigma_y(j)}$$

Here, μ is the mean of the marginal row or col probabilities p, while σ is the standard deviation of the marginal row or col probabilities p, with

$$p_x(i)=\sum_{j=1}^{N_g}P(i,j)\qquad p_y(i)=\sum_{j=1}^{N_g}P(i,j)$$

CET1-w_5_GLRLM_RP

Therun percentage in the gray-level run-length matrix of textural features in the X_{HLL} images:

$$RP=\sum_{i=1}^{N_g}\sum_{j=1}^{N_r}\frac{P(i,j|\alpha)}{N}$$

T2-w_Max3D

The shape and size feature that describes the maximum three-dimensional tumor diameter in the original image. This was measured as the largest pairwise Euclidean distance between voxels on the surface of the tumor volume.

T2-w_3_fos_mean

The first-order statistics feature that describes the mean value of the intensity levels in T2-w images in the X_{LLH} images:

$$mean=\frac{1}{N}\sum_{i}^{N}X(i)$$

T2-w_4_fos_mean

The first-order statistics feature that describes the mean value of the intensity levels in T2-w images in the X_{LHH} images. The formula is the same as has been shown for ***T2-w_3_fos_mean***.
